# Differences in Diagnosis and Management of Hypertensive Urgencies and Emergencies According to Italian Doctors from Different Departments Who Deal With Acute Increase in Blood Pressure—Data from Gear (Gestione Dell’emergenza e Urgenza in ARea Critica) Study

**DOI:** 10.3390/jcm11112986

**Published:** 2022-05-25

**Authors:** Francesca Saladini, Costantino Mancusi, Fabio Bertacchini, Francesco Spannella, Alessandro Maloberti, Alessandra Giavarini, Martina Rosticci, Rosa Maria Bruno, Giacomo Pucci, Davide Grassi, Martino Pengo, Maria Lorenza Muiesan

**Affiliations:** 1 Department of Medicine, University of Padova, 35128 Padova, Italy; 2 Cardiology Unit, Cittadella Town Hospital, Via Casa di Ricovero 40, 35013 Cittadella, Italy; 3 Hypertension Research Center, Department of Advanced Biomedical Science, Federico II University of Naples, 80131 Naples, Italy; 4 Internal Medicine, University of Brescia-ASST Spedali Civili di Brescia, 25121 Brescia, Italy; fabiobertacchini@yahoo.it (F.B.); marialorenza.muiesan@unibs.it (M.L.M.); 5 Internal Medicine and Geriatrics, IRCCS-INRCA, 60127 Ancona, Italy; fspannella@gmail.com; 6 Department of Clinical and Molecular Sciences, University “Politecnica delle Marche”, 60126 Ancona, Italy; 7 Cardiology 4, ASST Niguarda Hospital, 20121 Milan, Italy; a.maloberti@campus.unimib.it; 8 School of Medicine and Surgery, Milano-Bicocca University, 20126 Milan, Italy; 9 Cardiology Unit, Luigi Sacco Hospital, ASST Fatebenefratelli Sacco, 20154 Milan, Italy; alegiavarini@hotmail.com; 10 Medicine and Surgery Sciences Department, Alma Mater Studiorum University of Bologna, 40126 Bologna, Italy; mrosticci@yahoo.com; 11 Department of Clinical and Experimental Medicine, University of Pisa, 56126 Pisa, Italy; rosam.bruno@gmail.com; 12 Internal Medicine, Department of Medicine, University of Perugia, Terni University Hospital, 06132 Perugia, Italy; giacomo.pucci@gmail.com; 13 Department of Internal Medicine and Public Health, University of Aquila, 67100 L’Aquila, Italy; davide.grassi@univaq.it; 14 Department of Cardiovascular, Neural and Metabolic Sciences, IRCCS Istituto Auxologico Italiano, 20100 Milan, Italy; martinopengo@gmail.com

**Keywords:** hypertensive emergency, hypertensive urgency, treatment, blood pressure

## Abstract

Background: Diagnosis and treatment of hypertension emergency (HE) and urgency (HU) may vary according to the physicians involved and the setting of the treatment. The aim of this study was to investigate differences in management of HE and HU according to the work setting of the physicians. Methods: The young investigators of the Italian Society of Hypertension developed a 23-item questionnaire spread by email invitation to the members of Italian Scientific societies involved in the field of emergency medicine and hypertension. Results: Six-hundred and sixty-five questionnaires were collected. No differences emerged for the correct definitions of HE and HU or for the investigation of possible drugs that may be responsible for an acute increase in BP. The techniques used to assess BP values (*p* < 0.004) and the sizes of cuffs available were different according to the setting. Cardiologists more frequently defined epistaxis (55.2% *p* = 0.012) and conjunctival hemorrhages (70.7%, *p* < 0.0001) as possible presentation of HE, and rarely considered dyspnea (67.2% *p* = 0.014) or chest pain (72.4%, *p* = 0.001). Intensive care (IC) unit doctors were more familiar with lung ultrasound (50% *p* = 0.004). With regard to therapy, cardiologists reported the lowest prescription of i.v. labetalol (39.6%, *p* = 0.003) and the highest of s.l. nifedipine (43.1% *p* < 0.001). After discharge, almost all categories of physicians required home BP assessment or referral to a general practitioner, whereas hypertensive center evaluation or ambulatory BP monitoring were less frequently suggested. Conclusion: Management and treatment of HE and HU may be different according to the doctor’s specialty. Educational initiatives should be done to standardize treatment protocols and to improve medical knowledge.

## 1. Introduction

Arterial hypertension is a major public health problem involving more than 1 billion persons worldwide [1,2], and it is closely correlated to cardiovascular diseases, which are the leading cause of death all around the world [3]. About one to two percent of patients affected by arterial hypertension will developed acute blood pressure (BP) elevation during their lifetimes [4], which may evolve into hypertensive emergency (HE) or hypertensive urgency (HU). These may have a one-year death rate greater than 70% if left untreated [5]. HE is a condition characterized by high BP, usually above 180 mmHg for systolic BP (SBP) and/or above 110 mmHg for diastolic BP (DBP) associated with new onset or worsening of acute organ damage. The second condition, HU, differs from the first one by the absence of acute organ damage [5,6,7]. While treatment strategies for chronic hypertension are well codified in the most recent international guidelines [5,7,8], few data are available for diagnosis and treatment of acute BP elevation [9,10]. In particular, epidemiological data on prevalence, clinical features and protocols available for management and treatment of patients referred to emergency departments (Eds) for acute elevation of BP are limited, in spite of their relevance from a public health perspective [11,12,13]. Recently, two Italian research groups faced this problem when investigating prevalence and clinical presentations in some specific centers across Italy [14,15], whereas the Young Investigators of the Italian Society of Hypertension proposed the Gestione dell’Emergenza e urgenza in Area critica (GEAR) project, a survey aimed at evaluating the awareness, diagnosis and treatment of HE and HU in Italy [16]. In particular, this survey highlighted some critical aspects involving equipment available to face HE and HU, and some discrepancies still existing for the treatment of these conditions in clinical practice [15,16], particularly in comparison to what is currently suggested by most recent guidelines [5,7]. Moreover, some interesting differences emerged when the management and treatment of these two conditions were examined according to different macro-areas (north, center and south of Italy), and ED physicians’ practices were compared to those of other specialists [16]. As the problem of acute BP rise is peculiar not only to Eds, since it usually occurs (or relapses) in patients managed by the general practitioners, or in patients already hospitalized either in the intensive care units (ICU) (typically coronary or stroke unit) or in the medicine wards, a heterogenous group of medical specialists usually deals with these patients, and the approach and treatment given may be dependent on the scientific background of the specialists. In the GEAR project, we also explored the clinical practices of different specialists, according to different geographical areas. The aim of the present study was to complete previous findings while exploring the different management and treatment of these medical problems according to specialties.

## 2. Materials and Methods

On 22 December 2017, the “Young Investigator Researching Group” of the Italian Society of Hypertension, in collaboration with other Italian Scientific Societies involved in the diagnosis and treatment of HE and HU (Italian Society of Emergency and Urgency Medicine (SIMEU), Coordinating group of the residents in Emergency and Urgency Medicine (CoSMEU), Academy of Emergency Medicine and Care (AcEMC)), opened an online survey on the diagnosis, management and treatment of HE and HU in target areas. The survey remained accessible through a web platform until 15 March 2018 (before the ESC/ESH 2018 guidelines’ release [7]). The members of the scientific societies mentioned above were invited by an email describing the purpose of the initiative to respond to the questionnaire through a direct link to a web platform. The survey was developed according to the 2013 ESH/ESC hypertension guidelines [17], as the latest 2018 ESC/ESH Guidelines [7] were not yet published at the time of questionnaire’s realization and release, as mentioned above. The participation was voluntary and confidential, and each responder could withdraw at any point. The questionnaire consisted of 23 different items regarding diagnosis, management, treatment of HU and HE, methodology for BP measurements, eventual patient admission and follow-up after discharge. Some were multiple choice questions, and others required only one answer. The full detailed description of the questionnaire is available in the supplementary data and reported elsewhere [16]. At the beginning of the questionnaire, participants were invited to identify themselves as residents or consultants and according to their departments: EDs and/or emergency and urgency medicine (EUM) wards; cardiology units; internal medicine; IC or stroke units. According to the Italian health system, residents are students with degrees in Medicine and Surgery who are attending specialization school, whereas consultants are those who have already attended specialization school, have passed specialization exams and are regularly employed. Moreover, according to the Italian health system, ED doctors may be doctors with different specializations who are employed in the ED ward, whereas emergency and urgency doctors are those who attended the specific specialization school of emergency and urgency medicine and may be employed in the ED ward, or in internal medicine or an EUM ward (where these units are present).

### Statistical Analysis

We report continuous variables as mean ± standard deviation (SD). Categorical variables are expressed as frequencies and percentages. The chi-square distribution was used to compare categorical variables. Differences between continuous variables were calculated via Student’s *t*-test. In the Results, data are presented as comparisons between residents and consultants and between the five different departments. Data were calculated using Excel, Systat version 11, 12 (SPAA Inc., Evanston, IL, USA) and Statistical Package for the Social Sciences version 13 (SPSS Inc., Chicago, IL, USA). A two-tailed *p*-value < 0.05 was considered statistically significant in all analyses.

## 3. Results

Six-hundred and sixty-five questionnaires were collected (rate of response 55.42%): 59.7% from EDs, 22% from EUM, 8.7% from Cardiology Units, 5.7% from Internal Medicines and 3.9% from IC or Stroke units; 73.8% of the responders were consultants and 26.2% were residents.

The recognition of the correct definitions HE and HU according to the different specialties and the different qualification levels; and the rate of persons who investigated the use of illegal drugs, COX-1, COX-2 and steroids, which may be responsible for an acute BP increase, are presented in Table 1. Additionally, the attitudes toward the use of anti-anxiety drugs to reduce an acute BP increase, due to anxiety, from real HE or HU, are described in Table 1. Doctors working in different settings and at different career stages demonstrated the same knowledge of the definitions of HE and HU and had the same attitudes toward investigation of medications that may be responsible for an acute BP elevation (Table 1).

The techniques used by different specialists to measure BP are described in Table 2, and the availability of various sizes of cuffs by department are reported in Table 3. The majority of specialists detected BP from two to several times within the frame of several minutes and usually on both arms, unattended BP was rarely used and invasive detection was the exclusive prerogative of IC or stroke unit doctors (Table 2).

Almost all working settings declared having standard cuffs; large cuffs were quite extensively used except in cardiology (only 55.2%). Small cuffs were quite available in ED, EUM, internal medicine and IC or stoke units, but were not so frequent again in cardiology wards (34.5%). Extra-large cuffs were available in almost half of EDs, internal medicine departments and IC or stroke units, but were rare again in cardiology and in EUM (Table 3). Some interesting differences emerged regarding investigating signs and symptoms recognized by physicians as suggestive of acute organ damage due to an acute increase in BP (Table 4). Of note, cardiologists were prone to consider epistaxis and conjunctival hemorrhages as typical signs of acute BP elevation, and less frequently considered dyspnea and chest pain as possible signs of acute BP elevation. Residents and consultants did not show significant differences except for visual disturbances, which were considered possible signs of acute BP elevation more frequently by the former compared to the latter.

The laboratory and/or instrumental assessments required by different physicians to detect acute organ damage due to acute rise of BP are presented in Table 5. Some types of exams were used by physicians more commonly in some departments than others. For example, lung ultrasound was more frequently required by IC or ED doctors but very rarely by cardiologists (the same was true for chest radiography). Almost all responders required ECG and renal function tests, whereas fundoscopy was very rarely asked for, except by cardiologists. No significant difference emerged from comparisons between residents and consultants.

The therapeutic approach undertaken by doctors to reduce acute BP differed according to the departments they were in. In patients presenting with a HU, residents more frequently prescribed oral therapy as compared to consultants (76.9% vs. 68.6% vs. *p* = 0.05), but the treatment more frequently chosen for HE was intravenous (i.v.) drugs in both groups (96.5% among residents and 93.8% among consultants, *p* = n.s.). With regard to the different approaches of different specialists, similar rates of physicians correctly treated HU with oral drugs (71.8% among ED doctors, 65.1% among EUM doctors, 65.5% among cardiologists, 73.0% among internal medicine doctors and 96.1% among IC or stroke unit doctors; *p* = n.s.) and HE with i.v. drugs (94.7% among ED doctors, 96.6% among EUM doctors, 91.4% among cardiologists, 92.1% among internal medicine doctors and 96.1% among IC or stroke unit doctors). The medications preferred by department are listed in Table 6. The most frequently preferred drug in all categories was i.v. nitroglycerine. The use of i.v. nicardipine and i.v fenoldopam was very rare among all specialist categories and among residents and consultants. I.v. sodium nitroprussiade was quite rare except among cardiologists, but cardiologists less frequently prescribed i.v. labetalol and i.v. urapidil. The prescription of s.l. nifedipine was quite rare except among cardiologists, receiving 43.1% support. The use of i.v was less frequent among IC or stroke unit doctors, and that of i.m. clonidine and oral captopril was rare among EUM doctors. I.v. furosemide was widely used by all, though with a higher frequency among cardiologists (Table 6).

**Figure 1:**
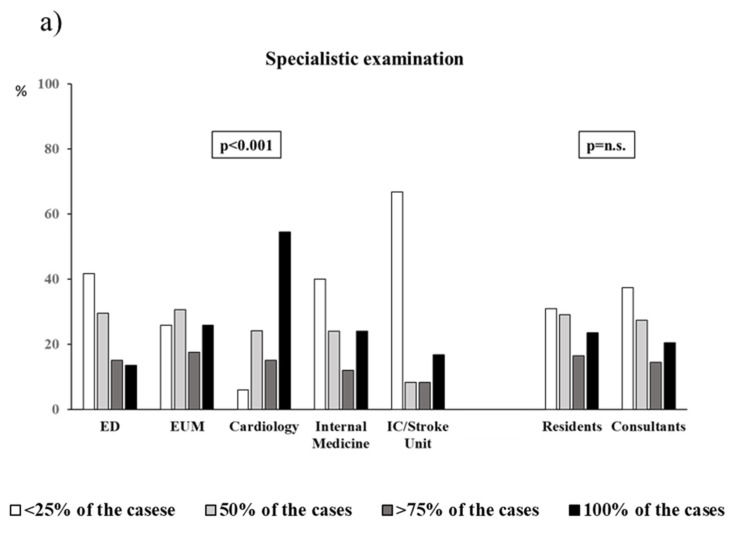

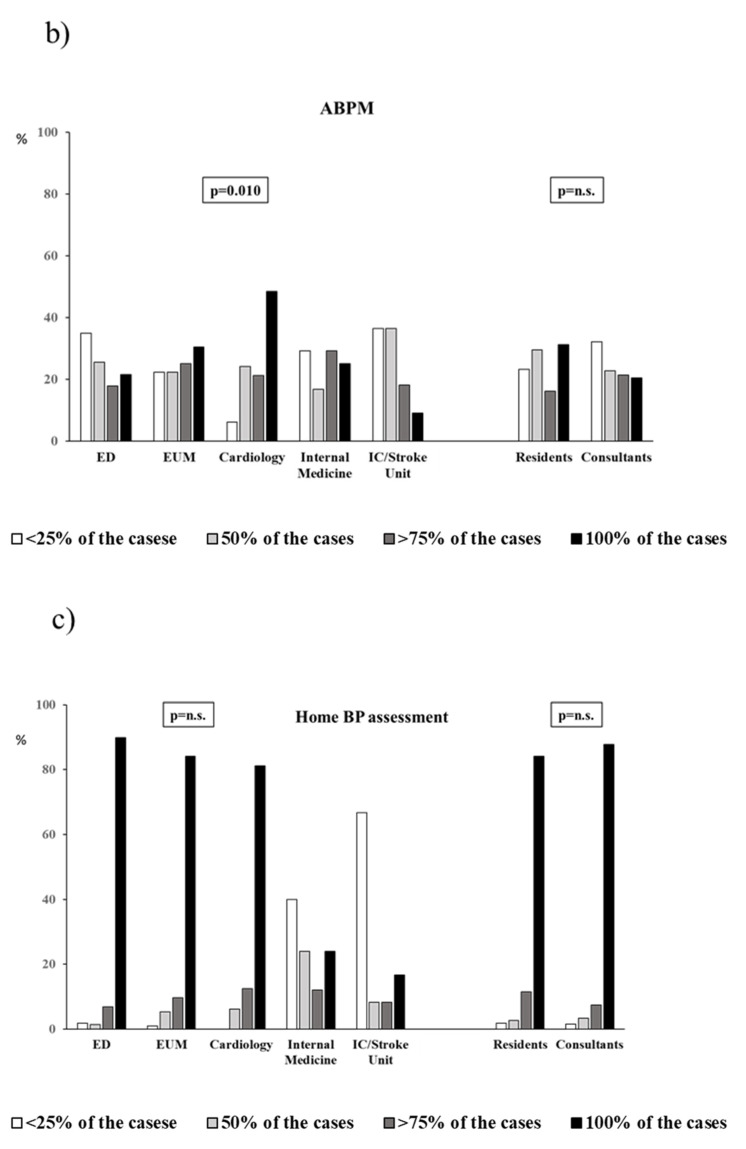

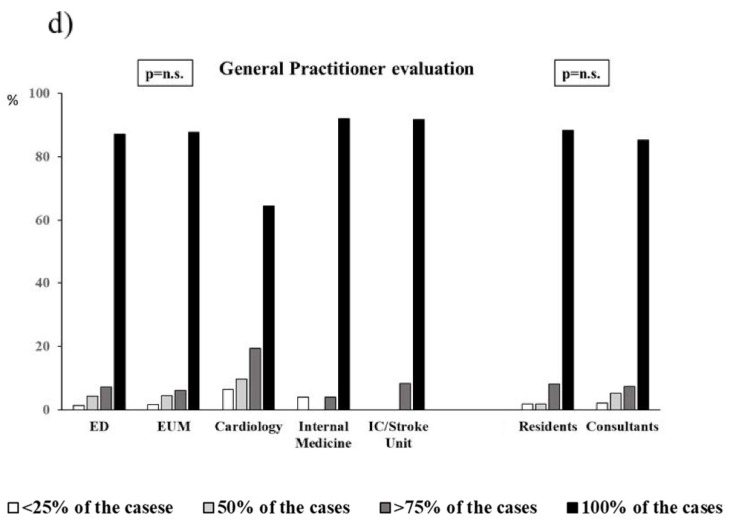

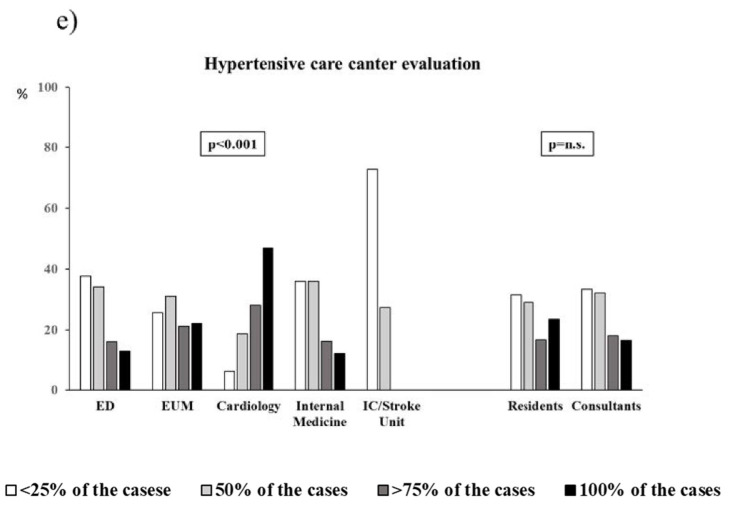
Prescribed follow-up visits after hospital discharge by department and qualification level. (**a**) Prescription of specialistic examination; (**b**) Prescription of Ambulatoory Blood Pressure Monitoring; (**c**) Prescription of home blood pressure monitoring; (**d**) Prescription of general practitioner evaluatiion; (**e**) Prescription of hypertensive care center evaluation. ED emergency Department; EUM Emergency and Urgency Medicine; IC Intensive Care.

After discharge form hospital, the follow-ups suggested are described in Figure 1 according to department and qualification level.

Almost all categories required general practitioner re-evaluation and home BP assessment (panel c and d). Specialistic examination (referral to a dedicated hypertension specialist center) was required more frequently by cardiologists (54.4% required it in 100% of cases) and very rarely among IC or stroke unit doctors (66.7% of them required it in less than 25% of cases) (panel a). Again, 24 h ambulatory blood pressure monitoring was more frequently suggested by cardiologists after discharge (48.5% required it in 100% of cases) and less frequently by IC or stroke unit and ED physicians (36.4% and 34.9%, respectively, required it in less than 25% of cases) (panel b); as was hypertensive center evaluation: 46.9% of the cardiologists suggested it in 100% of cases, whereas 72.7% of the IC or stroke unit doctors suggested it in less than 25% of cases (panel e). No significant difference was observed in the comparison between residents and consultants.

## 4. Discussion

According to the literature, HE should be managed in a similar way by the different specialists, possibly with standardized protocols, and always in the hospital setting with i.v medications; and HU should be managed outside of hospital or by emergency services, and usually with oral drugs. However, often this is not the real practice of HE and HU treatment among a great variety of different physicians. Thus, the main aim of the present study was to describe and characterize differences in classifying, managing and treating HU and HE among different specialists that deal with it. We addressed our specific aim by investigating potential discrepancies between consultants and residents, and among ED and EUM physicians, cardiologists, internal medicine doctors and IC or stroke unit doctors, because potential differences in the management of HU and HE could reflect different levels of awareness of the problem. Indeed, based on different perceptions of particular aspects related to the care of patients with HU and HE, the answers of various specialists could reveal differential cultural backgrounds that influence the decision-making process.

Our results showed that for the vast majority of main decisional knots, all the categories exhibited substantial similarity in diagnostic and therapeutic approaches. For instance, very similar approaches to the problems of HE and HU were observed for EUM and ED physicians, probably due to the fact that some of the scientific background of doctors working in EUM and EDs is very similar, and according to the organization of scientific societies in Italy, there are several occasions for comparison and dialogue during scientific meetings between these two categories of specialists. However, the survey highlighted some interesting discrepancies that deserve to be discussed. Of note, regarding investigating signs and symptoms suspicious of target organ damage due to acute BP elevation, we observed that cardiologists were less likely to consider dyspnea and chest pain as possible presentations of an acute increase in BP, whereas they become suspicious of signs and symptoms that are not peculiar, such as epistaxis and conjunctival hemorrhages. This was probably due to the diseases that these specialists are confident at dealing with: the symptom “chest pain” is probably referred to as a myocardial infarction and “dyspnea” as pulmonary oedema by cardiologists, perhaps making them forget that they may also be signs of acute organ damage due to HE. With regard to examinations required to investigate possible target organ damage due to BP, we noted that IC physicians had the highest use of lung ultrasound and chest X-rays in the acute setting. Again, this is probably related to their clinical experience, as these exams are useful to diagnose or exclude the presence of pulmonary oedema. According to the most recent literature, the examination of the lung with ultrasound may give to the clinicians large amounts of information in a single image, including that required in the identification of pulmonary diseases, pleural effusion and extravascular lung water [18]. In particular, in patients with acute dyspnea, a diffuse B profile pattern can be attributable to traditional interstitial syndrome conditions, such as acute cardiogenic pulmonary oedema [19,20].

Our study allowed us to recognize important differences in HE and HU management according to different working departments, strongly suggesting that a specific pathway of care needs to be implemented in each department. In particular, fundoscopy is more frequently prescribed by cardiologists than doctors in all other specialties, which do not frequently prescribe the examination, despite the current recommendation [6]. On the other hand, the use of lung ultrasound, which may be of great help in the diagnosis of HF, is rarely considered by cardiologists compared to doctors with other specialties. We observed that residents prescribed oral drug treatment more frequently for HU compared to consultants, as suggested by guidelines [5,6,7]. On the contrary, for the treatment of HE, residents more commonly used i.v. drugs, particularly nitroglycerine and nicardipine, though the differences were not statistically significant, and used oral captopril less often, compared to consultants—again in line with the literature [5,6,7]. Such differences in the management of individuals with HU and HE could reflect a different approach to sources of information for standard of care and an increased level of adherence to the most recently published guidelines [5,6,7]. Indeed, in the latest version of guidelines, there is great emphasis on the recommendation not to give i.v. drugs to patients suffering an HU, and in favor of the use of i.v. up- or down-titrating drugs for the management of HE [5,6,7]. Surprisingly, residents and consultants did not show any difference in the prescription of s.l. nifedipine, which has been the subject of great concern when used during hypertensive crises, because, even if orally given, it could be related to an increased risk of an abrupt BP decrease, which is associated with a high risk of uncontrolled systemic tissue hypoperfusion [21]. According to the literature, several cases of acute BP decrease after s.l. nifedipine administration have occurred, and papers have reported the dramatic consequences of such an erroneous prescription, including cerebrovascular ischemia, severe hypotension, acute myocardial infarction, conduction disturbances, fetal distress and death [22,23,24,25,26,27]. Unfortunately, this evidence did not come from large randomized clinical trials, but from a few small trials, though they clearly depicted the dramatic results of this incorrect prescription. Indeed, it has been recommended by several papers that the use of instant release formulations of nifedipine in the management of acute BP elevation should be either totally prohibited or severely restricted [28,29]. However, despite clear recommendations in several guidelines [5,6,7], we found that almost a quarter of responders in both groups (20.5% of consultants and 25.1% among residents) would prescribe s.l. nifedipine; similar rates were observed among EUM (17.8%) and ED doctors (22.7%). Surprisingly, the rate reached 43.1% among cardiologists, once again highlighting a knowledge gap which needs to be filled among a number of specialists. We might speculate that the larger use of SL nifedipina among cardiologists is related to the larger availability of the drug within the cardiology departments compared to others. Of note, cardiologists less frequently used i.v. labetalol and i.v. urapidil, in favor of more use of i.v. furosemide and i.v. nitroprusside, indicating greater familiarity with these kinds of drugs that they more frequently use in their daily clinical practice as first choice medicines for several diseases, such as pulmonary oedema and acute heart failure.

Another interesting fact raised by the survey was the high level of oral captopril use. The use of this drug may limit the possibility of performing investigations for a secondary form of hypertension due to this addition of a renin–angiotensin–aldosterone blocker [30]. According to Endocrine Society Practice Guideline, the use of this drug may reduce aldosterone and greatly increase renin, confounding the detection of a case of primary aldosteronism (secondary hypertension) [31]. Other interesting matter of debate is the optimization of the out-of-hospital care of subjects with uncontrolled hypertension after ED discharge. As highlighted by the survey, there was sub-optimal referral to hypertension specialists and also to specialist evaluations, along with a lack of prescribing ambulatory BP monitoring to confirm the state of the BP, by almost all groups of specialists except for cardiologists, who showed the highest rate of hypertension specialist referral and ambulatory BP monitoring. Our survey highlighted that a great number of the participants, especially ED and EUM doctors, did not consider ambulatory BP monitoring or a specialist evaluation after patient discharge, and this has resulted for the patients in absence of the chance to be adequately followed by an expert in the field, and probably frequent relapsing of re-hospitalization and/or uncontrolled BP. As documented by Patel KK et al. [32], there is no difference among patients with HU referred to ED or sent home, in terms of improved outcome or controlled BP. The only difference observed was in the follow-up: those patients who had a designated primary care physician had a better average 6-month follow-up. This evidence confirms the importance of having a strictly designed program for patients with uncontrolled BP; otherwise, as documented by the paper, there will be no success in terms of preventing major adverse cardiovascular events or improving BP control [32]. The importance of regular follow-up for these patients was well emphasized by the 2018 ESC/ESH guidelines [7], which recommended, after discharge from hospital with safe and stable BP and oral therapy, frequent—at least monthly—visits to a specialized setting until the optimal target BP is achieved, and a long-term specialist follow-up thereafter.

## 5. Strengths and Limitations

The main strength of this analysis was the good proportions of responses from among the main specialties that face HE and HU, and there was a good distribution between residents and consultants. Of course, the majority of physicians came from Eds, which were the main targets of our survey. We collected only a small number of responders working in internal medicine (36 physicians) and IC or stroke units (only 26 doctors). Moreover, the survey was not spread to gynecologists, which in Italy manage all the diseases dealing with pregnancy, so data on eclampsia or severe preeclampsia are lacking in our analysis. We are conscious that this paper has some limitations. First of all, the analyses being based on an online survey allowed us to perform only a descriptive analysis, and we cannot provide evidence of causation. The rate of response was not very high, and this might express the lack of familiarity with this issue and reticence to discussing it. Moreover, the result of the questionnaire may not be representative of the wider community of Italian physicians that deals with HE and HU. In particular, we cannot compare the different rates of response according to specialization, due to the fact that the questionnaire was spread by the scientific societies listed above to their members, and then thought them to their colleagues working in the same hospital. In addition, the answers given to an email survey may reflect the “ideal” practice according to the literature and guidelines and not the real everyday clinical practice. However, as the questionnaire was anonymous, and this makes us quite confident that the responses represent the true everyday management of HE and HU. Moreover, we are not able to distinguish between physicians working in rural or urban areas, though the access to some diagnostic facilities and pharmacological treatments, and connections with hypertension center/hypertension specialists, may be different in these two different conditions. The ability to update knowledge may also differ. Due to the nature of the questions proposed in the survey, which mainly referred to “acute increase/elevation of BP” we were not able to distinguish results between HE and HU (a detailed description of the entire questionnaire can be found in our previous paper [16]). Finally, the last limitation was that the survey was spread before the release of the 2018 ESC/ESH hypertension guidelines [7], and according to the available guidelines at that time [17], the threshold with which to identify HE and HU was SBP > 180 mmHg and/or DBP > 120 mmHg, though we are quite confident that the higher BP levels of the previous guidelines did not influence the responses of the doctors. Our results highlight some difference in the clinical management of HE and HU between resident and consultants. However, in some hospital settings, residents are not always allowed to make independent treatment decisions.

## 6. Conclusions

One of the key points of this investigation was the discrepancies in the management of HE and HU due to the different scientific backgrounds of the doctors in various departments. This highlighted the need to share and discuss the last updates on the management and treatment of HE and HU to different scientific societies, in order to standardize the management of patients experiencing these maladies among the vast majority of physicians. This was confirmed by the high percentage of participants who were in favor of updating meetings (88.7% of specialists). Further evidence should be found on if scientific meetings are the best way to spread the knowledge and to improve the management and treatment of HE and HU.

## Figures and Tables

**Table 1:** Rates of persons who identified the correct definitions of HE and HU; who use anti-anxiety drugs to counter an acute BP rise; and who investigated the use of illegal drugs, COX-1, COX 2 and steroid drugs, according to specialty.

Correct Definition	EUM *n* = 146	ED *n* = 397	Cardiology *n* = 58	Internal Medicine *n* = 38	IC or Stroke unit *n* = 26	*p*	Residents *n* = 174	Consultants *n* = 491	*p*
HU, %	76.7	81.4	82.8	89.5	88.5	n.s.	83.2	80.4	n.s.
HE, %	91.8	88.2	84.5	97.4	92.3	n.s.	92.5	88.2	n.s.
Use of anti-anxiety drugs	37.7	34.8	34.5	34.3	26.9	n.s.	36.8	34.4	n.s.
Investigation of									
Illegal drugs, %	95.9	98.5	93.1	97.4	100	n.s.	97.7	97.4	n.s.
COX-1, %	45.9	45.1	44.8	47.4	50	n.s.	45.6	45.3	n.s.
COX-2, %	47.3	43.1	43.1	44.7	38.5	n.s.	46.8	43.2	n.s.
Steroids, %	80.8	82.1	89.6	78.9	76.9	n.s.	84.8	81.6	n.s.

Data are presented as percentages. EUM, emergency and urgency medicine; ED, emergency department; IC, intensive care; HU, hypertensive urgency; HE hypertensive emergency. *p* values are expressed as χ^2^; n.s. not statistically significant, *p* > 0.05.

**Table 2:** Techniques used to detect BP levels by department and qualification level.

Techinque	EUM *n* = 146	ED n = 397	Cardiology *n* = 58	Internal Medicine *n* = 38	IC or Stroke Unit *n* = 26	*p*	Residents *n* = 174	Consultants *n* = 491	*p*
One sigle detection	16.4	22.9	22.4	15.8	34.6	0.004	23.4	20.7	n.s.
Two detection within 1–3 min	34.2	34.5	37.9	26.3	26.9	0.004	33.9	34.4	n.s.
Several detections in both arms	45.9	37.8	37.9	47.4	30.8	0.004	35.7	40.8	n.s.
Unattended office BP	2.0	3.5	0	2.6	0	0.004	2.3	3.0	n.s.
Several detection or invasive (intra-arterial) detection	0	1.2	1.7	2.6	7.7	0.004	3.5	0.6	n.s.
It depends according to the different patient	1.4	0	0	5.3	0	0.004	1.2	0.4	n.s.

Data are presented as percentages. EUM, emergency and urgency medicine; ED, emergency department; IC, intensive care; BP, blood pressure; *p* values are expressed as χ^2^; n.s. not statistically significant (*p* > 0.05).

**Table 3:** Availability of different sizes of cuffs for correct detection of office BP by department and qualification level.

Type of Cuff	EUM *n* = 146	ED *n* = 397	Cardiology *n* = 58	Internal Medicine *n* = 38	IC or Stroke Unit *n* = 26	*p*	Residents *n* = 174	Consultants *n* = 491	*p*
Small, %	62.3	57.9	34.5	63.1	53.8	0.020	59.6	56.0	n.s.
Standard, %	92.5	94.5	93.1	92.1	100	n.s.	94.7	93.6	n.s.
Large, %	76.7	77.3	55.2	78.9	84.6	0.021	81.9	73.3	n.s.
Extra large, %	31.5	41.6	27.6	44.7	46.1	n.s.	32.7	40.4	n.s.
Universal, %	26.7	31.5	36.2	26.3	30.8	n.s.	28.6	31.6	n.s.

Data are presented as percentages. EUM, emergency and urgency medicine; ED, emergency department; IC, intensive care; small, cuff <24 cm in diameter; standard, cuff 24–32 cm in diameter; large, 32–42 cm in diameter; extra-large > 42 cm; universal; cuff suitable for different ranges of arms from small to large. *p* values are expressed as χ^2^; n.s. not statistically significant, *p* > 0.05.

**Table 4:** Signs and symptoms recognized as suggestive of acute organ damage due to BP increase, by department and qualification level.

Sign/Symptom	EUM *n* = 146	ED *n* = 397	Cardiology *n* = 58	Internal Medicine *n* = 38	IC or Stroke Unit *n* = 26	*p*	Residents *n* = 174	Consultants *n* = 491	*p*
Epistaxis, %	32.2	33.2	55.2	29.0	26.9	0.012	34.5	34.2	n.s.
Visual disturbances, %	90.4	88.9	93.1	84.2	100	n.s.	93.6	88.5	0.05
Tinnitus, %	41.8	35.0	50.0	42.1	34.6	n.s.	43.3	37.4	n.s.
Dyspnea, %	84.2	84.1	67.2	78.9	92.3	0.014	79.5	83.1	n.s.
Headache, %	84.2	80.6	86.2	86.8	76.9	n.s.	81.9	82.3	n.s.
Conjunctival haemorrhages, %	41.1	38.5	70.7	39.5	26.9	<0.001	47.4	39.5	n.s.
Dizziness, %	53.4	50.9	60.3	47.4	50	n.s.	49.7	52.6	n.s.
Chest pain, %	91.1	91.2	72.4	84.2	88.5	0.001	87.7	89.3	n.s.

Data are presented as percentages. EUM, emergency and urgency medicine; ED, emergency department; IC, intensive care; *p* values are expressed as χ^2^. n.s. not statistically significant (*p* > 0.05).

**Table 5:** Tests available and prescribed to identify acute target organ damage due to an acute rise in BP, by department and qualification level.

Test	EUM *n* = 146	ED *n* = 397	Cardiology *n* = 58	Internal Medicine *n* = 38	IC or Stroke Unit *n* = 26	*p*	Residents *n* = 174	Consultants *n* = 491	*p*
Fundoscopy, %	22.6	25.4	48.3	28.9	30.8	0.006	31.0	26.9	n.s.
TTE, %	63.7	65.7	67.2	52.6	76.9	n.s.	66.7	64.5	n.s.
Renal function (creatinine), %	93.1	91.2	91.4	89.5	88.5	n.s.	93.0	91.2	n.s.
Urinary protein, %	50.0	54.2	63.8	52.6	73.1	n.s.	56.1	56.0	n.s.
Brain CT scan, %	66.4	56.7	39.6	50.0	61.5	n.s.	53.2	58.5	n.s.
BNP/NT-pro-BNP, %	42.5	34.3	31.0	36.8	42.3	n.s.	38.0	36.5	n.s.
Markers of cardiomyocyte necrosis, %	67.1	66.2	63.8	63.2	69.2	n.s.	70.1	65.6	n.s.
Chest radiography, %	54.8	49.9	24.1	68.4	73.1	0.001	51.5	50.2	n.s.
ECG, %	97.9	97.7	93.1	92.1	100	n.s.	97.4	97.1	n.s.
Lung ultrasound, %	33.6	43.8	17.2	36.8	50.0	0.004	35.1	41.0	n.s.

Data are presented as percentages. EUM, emergency and urgency medicine; ED, emergency department; IC, intensive care; TTE, transthoracic echocardiography; CT, computed tomography; BNP, brain natriuretic peptide; ECG, electrocardiogram; *p* values are expressed as χ2; n.s. not statistically significant (*p* > 0.05).

**Table 6:** Drugs administered to reduce an acute increase in BP, by department and qualification level.

Drugs	EUM *n* = 146	ED *n* = 397	Cardiology *n* = 58	Internal Medicine *n* = 38	IC or Stroke Unit *n* = 26	*p*	Residents *n* = 174	Consultants *n* = 491	*p*
i.v. Sodium Nitroprusside, %	16.4	19.1	31.0	15.8	19.2	0.05	18.1	19.9	n.s.
i.v. Nitroglycerine, %	79.4	79.1	74.1	78.9	88.5	n.s.	80.1	78.6	n.s.
i.v. Labetalol, %	67.8	66.5	39.6	73.7	73.1	0.003	63.7	65.0	n.s.
i.v. Urapidil, %	65.1	67.8	22.4	71.0	46.1	<0.001	60.8	63.7	n.s.
i.v. or i.m. Clonidine, %	64.4	67.5	75.9	60.5	34.6	0.008	62.6	66.9	n.s.
Oral Captopril, %	30.8	52.6	46.5	60.5	38.5	<0.001	40.3	48.9	0.05
i.v. Furosemide, %	65.1	54.4	79.3	57.9	61.5	0.018	65.5	58.3	n.s.
s.l. Nifedipine, %	17.8	22.7	43.1	5.3	11.5	<0.001	25.1	20.5	n.s.
i.v. Nicardipine, %	4.8	4.0	8.6	2.6	3.8	n.s.	7.6	3.6	n.s.
i.v. Fenoldopam, %	2.7	6.8	10.3	2.6	3.8	n.s.	5.8	6.2	n.s.

Data are presented as percentages. EUM, emergency and urgency medicine; ED, emergency department; IC, intensive care; i.v., intravenous; i.m., intramuscular; s.l. sublingual. *p* values are expressed as χ^2^; n.s. not statistically significant (*p* > 0.05).

## Data Availability

Not applicable.
